# A robust multiplex immunoaffinity mass spectrometry assay (PromarkerD) for clinical prediction of diabetic kidney disease

**DOI:** 10.1186/s12014-020-09302-w

**Published:** 2020-10-20

**Authors:** Scott Bringans, Jason Ito, Tammy Casey, Sarah Thomas, Kirsten Peters, Ben Crossett, Orla Coleman, Holger A. Ebhardt, Stephen R. Pennington, Richard Lipscombe

**Affiliations:** 1 Proteomics International, Perth, Australia; 2 Sydney Mass Spectrometry, University of Sydney, Sydney, Australia; 3 Atturos, Dublin, Ireland

**Keywords:** Biomarkers, Assay development, Diabetic kidney disease, Immunoaffinity, Multiplex, MRM, Targeted mass spectrometry

## Abstract

**Background:**

PromarkerD is a novel proteomics derived blood test for predicting diabetic kidney disease (DKD). The test is based on an algorithm that combines the measurement of three plasma protein biomarkers (CD5L, APOA4, and IBP3) with three clinical variables (age, HDL-cholesterol, and eGFR). The initial format of the assay used immunodepletion of plasma samples followed by targeted mass spectrometry (MRM-LCMS). The aim of this study was to convert the existing assay into an immunoaffinity approach compatible with higher throughput and robust clinical application.

**Methods:**

A newly optimised immunoaffinity-based assay was developed in a 96 well format with MRM measurements made using a low-flow LCMS method. The stability, reproducibility and precision of the assay was evaluated. A direct comparison between the immunoaffinity method and the original immunodepletion method was conducted on a 100-person cohort. Subsequently, an inter-lab study was performed of the optimised immunoaffinity method in two independent laboratories.

**Results:**

Processing of plasma samples was greatly simplified by switching to an immunoaffinity bead capture method, coupled to a faster and more robust microflow LCMS system. Processing time was reduced from seven to two days and the chromatography reduced from 90 to 8 min. Biomarker stability by temperature and time difference treatments passed acceptance criteria. Intra/Inter-day test reproducibility and precision were within 11% CV for all biomarkers. PromarkerD test results from the new immunoaffinity method demonstrated excellent correlation (R = 0.96) to the original immunodepletion method. The immunoaffinity assay was successfully transferred to a second laboratory (R = 0.98) demonstrating the robustness of the methodology and ease of method transfer.

**Conclusions:**

An immunoaffinity capture targeted mass spectrometry assay was developed and optimised. It showed statistically comparable results to those obtained from the original immunodepletion method and was also able to provide comparable results when deployed to an independent laboratory. Taking a research grade assay and optimising to a clinical grade workflow provides insights into the future of multiplex biomarker measurement with an immunoaffinity mass spectrometry foundation. In the current format the PromarkerD immunoaffinity assay has the potential to make a significant impact on prediction of diabetic kidney disease with consequent benefit to patients.

## Background

Research to discover protein biomarkers for disease detection and progression by mass spectrometry has produced a wealth of putative markers, with a simple Pubmed search of “Protein Biomarker” returning more than 60,000 links for review articles alone. The translation of these discoveries to analytically validated tests and more importantly to clinically useful tests has been less than forthcoming [1, 2]. The hindrances include complexity of the tests, high costs of developing and running the protein based tests and reproducibility of the protein assay [3]. The current method of choice for clinical measurement of protein biomarkers is the enzyme-linked immunosorbent assay (ELISA), a technology in use for close to 50 years [4, 5] with the ability to scale to large numbers of relatively rapid tests [3, 6]. However, a major advantage that mass spectrometry can provide, particularly with regard to protein detection, is the ability to multiplex where panels of biomarkers are measured concurrently. The advent of increasingly sensitive mass spectrometers and the abundance of techniques for quantifying large proteome datasets has had the effect of producing panels of biomarkers that can be combined in a model to determine a disease state or progression. Examples of such tests are the FDA-cleared OVA1 diagnostic test that combines levels of 5 protein biomarkers to determine a score that assists a clinician in the assessment of the patient’s risk of ovarian cancer [7, 8] and the Vectra DA test that utilises 15 protein biomarkers and clinical variables to measure inflammation caused by rheumatoid arthritis [9]. A hybrid test that harnesses the selectivity of antibody capture and the multiplexing capability of multiple reaction monitoring (MRM) targeted mass spectrometry offers the potential to convert what may only be suitable as a ‘research’ test into an assay more suited to high throughput clinical applications.

Diabetic Kidney Disease (DKD) is a significant complication of diabetes with one in three adult diabetics having some degree of DKD [10]. The current diagnostic tests to monitor DKD are the urinary albumin:creatinine ratio (ACR) and the estimated glomerular filtration rate (eGFR) which is a blood test. These tests can independently determine a person’s kidney function, however they can provide conflicting diagnosis and have minimal predictive power to determine a patient’s disease progression [11].

PromarkerD is a test that can successfully predict 4 years in advance that a patient will develop chronic kidney disease (CKD) [12, 13]. It was developed using a proteomics biomarker discovery and validation workflow [14]. The output risk score of the test uses a panel of 3 protein biomarkers (Apolipoprotein A4 (APOA4), CD5 antigen-like (CD5L) and Insulin-like growth factor-binding protein 3 (IBP3)) measured by MRM mass spectrometry combined with three clinical variables (age, hdl-cholesterol and eGFR) in a predictive algorithm [13]. The workflow of the biomarker measurement involved immunodepletion of the top 14 abundant plasma proteins before diafiltration, reduction, alkylation, digestion and targeted mass spectrometry [14]. The process while suitable for a research test to validate and prove the utility of the biomarkers is not sufficiently cost-effective or rapid enough for a potential clinical application.

A new higher-throughput and more robust test was developed using an immunoaffinity bead-based approach with mass spectrometry detection. An affinity capture step allows the concentration of the target proteins while removing the remaining plasma proteome with a simple wash step. There already exists affinity based approved clinical protein biomarker tests [15–19] but these are for single protein measurements. The optimisation of PromarkerD to an immunoaffinity-LC-MRM assay provides the advantages of antibody selectivity with the multiplexing capability of MRM detection. Data from the optimised assay was compared with the original immunodepletion method on a cohort of 100 patient samples and an inter-lab comparison was also completed using the same cohort. The decrease in assay time, compatibility with a robotic handling platform in a 96 well format and the use of microflow LCMS detection dramatically improved the throughput and robustness of the PromarkerD assay.

## Methods

### Reagents

Chemicals were from Sigma unless otherwise stated. NP-40, Tris(2-carboxyethyl)phosphine hydrochloride (TCEP) from Thermofisher; Iodoacetamide (IAM) from Astral Scientific. Synthetic isotopically labelled peptides (AQUA, Sigma) were LEPYADQL-R[^13^C_6_,^15^N_4_) from APOA4 protein, LVGGDNL-C(CAM)-SG-R[^13^C_6_^15^N_4_] from CD5L protein, and FLNVLSP-R[^13^C_6_,^15^N_4_] from IBP3 protein. A standard reference plasma was created by combining EDTA plasma from three healthy vounteers before aliquoting and storage at − 80 °C.

### Clinical samples

All clinical plasma samples were provided by the Fremantle Diabetes Study (FDS), a longitudinal observational cohort [20]. EDTA (Ethylenediaminetetraacetic acid) plasma was collected from all patients after an overnight fast and stored at − 80 °C until required. The FDS protocol was approved by the South Metropolitan Area Health Service Human Research Ethics Committee. All subjects gave informed consent before participation.

### Antibody production

Monoclonal antibody targeting the biomarker APOA4 was developed by the Monash Antibody Technologies Facility (MATF, Monash University, Melbourne, Australia) and monoclonal antibodies targeting CD5L and IBP3 were developed by CDI laboratories (Mayaguez, Puerto Rico). The antibodies were developed exclusively for this project and will be available commercially once manufacturing standards and regulatory requirements are fulfilled. The hybridoma cell lines were used for the production of purified antibodies by the Monoclonal Antibody Facility of the Harry Perkins Institute of Medical Research (Perth, Australia), tested for specificity and provided for use.

### Bead-antibody production

Batches of magnetic bead-antibody conjugates were made from Dynabeads® (M-270 Epoxy beads, Thermofisher) according to the manufacturer’s instructions at a ratio of 50:1 beads:antibody. The bead-antibody conjugate batches (“Ab-beads”) were quality control tested and stored in PBS at a concentration of beads at 10 mg/mL at 2–8 °C until use. Long term stability testing indicates a shelf life of at least 5 months at 2–8 °C (data not shown).

### Standards and controls

A calibrator standard was prepared by combining the recombinant protein biomarkers APOA4, CD5L and IBP3 (Sino Biologicals) in phosphate buffered saline (PBS) at concentrations of 18.3, 0.686 and 0.178 µg/mL respectively. Once processed alongside plasma samples they represented effective plasma concentrations of 91.5, 3.45 and 0.892 µg/mL. The synthetic isotopically labelled peptides were used for signal normalisation. The standard reference plasma (stored at − 80 °C) was processed (4 separate aliquots) with every batch of samples to allow monitoring of assay performance and quality control (QC) measurements. The acceptance criteria for a batch of samples (96 well plate) was that the standard reference plasma biomarker concentrations interpolated from the calibrator standard were within 2 standard deviations of the rolling averages of the biomarker concentrations from all the batches for which data had been acquired. New batches of the calibrator standard were required across the study (4 batches in total) and these were tested alongside the previous batch using the reference plasma biomarker concentrations meeting the same passing criteria as above for both old and new batches of calibrator standard (all batches passed).

### Sample processing

Equal volumes of “Ab-beads” were pooled from the three individual stock solutions, then the liquid removed using a magnet to hold the beads. The beads were then resuspended in a volume of PBS to provide 150 µL volume of beads in each well corresponding to 120 µg of each antibody-bead conjugate per well. The following steps were carried out with a robotic handling system (Janus, Perkin Elmer) unless otherwise stated. The Ab-beads were transferred from a trough to a 96 well plate (2 mL round bottom, Thermofisher). The calibrator standard (N = 4 replicates, 50 µL), the reference plasma (N = 4 replicates, 10 µL) and the samples (10 µL) were added to the plate along with blanks (PBS, 50 µL) and a double blank (200 µL PBS, no Ab-beads). All plasma samples had an additional 40 µL of PBS added to make all final volumes to 200 µL. The plate was incubated at 37 °C for 90 min with intermittent shaking (Thermomixer, Eppendorf) to keep beads suspended in solution. The liquid was removed with the plate on magnet and the beads washed with 800 µL of 50 mM triethylammonium bicarbonate (TEAB), 150 mM NaCl, 0.1% NP-40 and then a further wash with 800 µL of 50 mM TEAB. After removing the liquid, the beads were resuspended in 56 µL of 50 mM TEAB, 5 mM TCEP containing 400 fmoles of each of three synthetic ^13^C^15^N labelled peptides (one for each target protein) and incubated at 55 °C for 20 min (Thermomixer, Eppendorf). To each well was added 6 µL of 100 mM IAM and incubated at room temperature (RT) in the dark for 20 min. Sequencing grade trypsin (Sigma, catalog no. 1418475001) was then added to each well (10 µL of 0.05 µg/µL in milliQ water) and incubated at 37 °C for 16 h. The solution was transferred into a clean 96 well plate (300 µL V bottom, Greiner) for LCMS analysis.

### Targeted (MRM) LCMS analysis

The system used at the development laboratory (Proteomics International) was a Shimadzu Prominence HPLC system with Loading pump (40µL/min) to load the sample onto a trap column and two Nano pumps operating at a combined 5 µL/min to provide the analytical gradient. The column was kept at 40 °C and the 5 µL/min flow directed to a QTRAP 5500 mass spectrometer (Sciex) equipped with a 50 micron electrode and grounding unit (Sciex) for the Turbo-V ion source. From the V bottom plate 25 µL of each sample for analysis was injected onto a MicroLC Guard Column C18 (Sciex) flowing isocratically at 2% (v/v) Acetonitrile, 0.1% (v/v) formic acid at 40 µL/min for 2 min. The flow was then directed from the microflow pumps (5 µL/min) through the guard column into a ChromXP C18, 3 µm 120 Å 300 micron ID, 5 cm column (Sciex) with a gradient of 10–40% acetonitrile, 0.1% formic acid over 2 min. The flow was ramped to 98% acetonitrile over 0.2 min, held at that concentration for 0.2 min before returning to the starting conditions of 10% acetonitrile, 0.1% formic acid over 0.1 min. The total runtime was 8 min. The mass spectrometry settings were as follows: Source Temp 250 °C; IonSpray Voltage 5500; Curtain Gas 25; Collision Gas Medium; GS1 and GS2 25; Entrance Potential 10; Collision Cell Exit Potential 14; Q1 Resolution Low; Q3 Resolution Low. The transitions are shown in Additional file 1.

### Data analysis

Raw data files were imported into Skyline (v 3.6, MacCoss Lab Software, University of Washington, Seattle, USA) and peaks integrated to provide peak areas for each of the peptides and their corresponding labelled version with signal to noise (S/N) > 5 for all peaks based on the Total Area and Total Background values provided by Skyline. The calibrator replicate ratios of unlabelled:labelled peptide peak area for each biomarker protein were averaged and the known concentration of the calibrator protein used to determine the concentrations of each protein in unknown samples. In this way the labelled peptide is used to normalise signal across all samples analysed while the calibrator protein standards provide the concentrations.

### Linearity, detection range, stability, reproducibility and precision testing

Linearity and detection range of the assay was demonstrated with a dilution series of the recombinant biomarker proteins in PBS that was processed and analysed as for plasma samples. To test parallelism between the recombinant protein and actual plasma samples a further experiment was performed with a dilution series of the recombinant proteins in PBS versus a dilution series of a reference plasma diluted with a 5% human serum albumin solution (Sigma) in PBS as a surrogate matrix.

To assess sample stability to freeze/thaw and other temperature variations the following experiments were carried out.

- Two duplicates of 3 independent plasma samples (stored at − 80 °C) were defrosted to 4 °C and then N = 3 samples were processed at 1 h versus the duplicate N = 3 samples left at 4° for 24 h before processing.
- Two duplicates of 3 independent plasma samples were defrosted with N = 3 kept at RT for 1 h before processing versus the duplicate N = 3 samples left for 24hrs at RT before processing.
- Three independent plasma samples were defrosted versus a set of N = 3 replicates that had two more additional freeze thaw cycles (at least 1 h frozen at − 80 °C before thaw) performed on them before processing.
- Previously injected replicates of processed plasma (N = 86) were left in the HPLC autosampler for a further 24 h after their initial analyses before being re-injected.
- The precision testing was achieved with N = 4 replicates of a reference plasma processed in a single batch for an intra-day comparison and then this was repeated over twenty separate days for an inter-day comparison.

### Cross assay comparisons

A cohort of 100 patient samples from the FDS cohort were analysed by both the original immunodepletion method and the new immunoaffinity method. The biomarker concentrations from each method were then compared using Bland Altman plot analysis to determine the agreement between the two assays [21]. The mean bias between the two methods for each biomarker was determined from the Bland Altman difference plot. Any bias observed was then adjusted for by applying the mean difference to the biomarker concentrations measured by immunoaffinity. A hypothesis test for equality (Student’s *t*-test) was used to assess whether there was a statistical difference between concentrations measured by the two methods. The null hypothesis was that the bias is equal to zero (i.e. there is no difference between the two methods), against the alternative hypothesis that it is not equal to zero. Significant test p-values resulted in rejecting the null hypothesis and concluding that the bias is different to zero. The PromarkerD risk scores were calculated for each patient sample from each analysis method, using the adjusted immunoaffinity concentrations. The two PromarkerD scores were then compared by scatter plot and the correlation between the two methods assessed. An allowable difference of 5% was used to assess the number of subjects with larger differences between the methods. The acceptable percentage of subjects within the 5% difference was set at > 90% of the cohort.

### Inter-laboratory comparisons

To assess whether peptide transitions and the LCMS method were transferable between laboratories a set of processed samples using the original immunodepletion method were split and run at two sites (Sciex 4000 QTRAP at the development laboratory and a Sciex 5500 QTRAP at Sydney Mass Spectrometry, University of Sydney).

To establish the clinical robustness of the immunoaffinity method 100 plasma samples were independently processed and analysed in the development laboratory and a third site (Atturos (Dublin, Ireland)). This comparison laboratory used an Agilent 1290 LC and 6495B mass spectrometer for detection. For comparison between laboratories biomarker concentrations were adjusted using the respective mean bias determined from Bland Altman Plot analysis, as described above. To be able to compare the risk predictions the comparison laboratory data was further adjusted to the immunodepletion concentrations as had already being done for the development laboratory values.

## Results

**Fig. 1:**
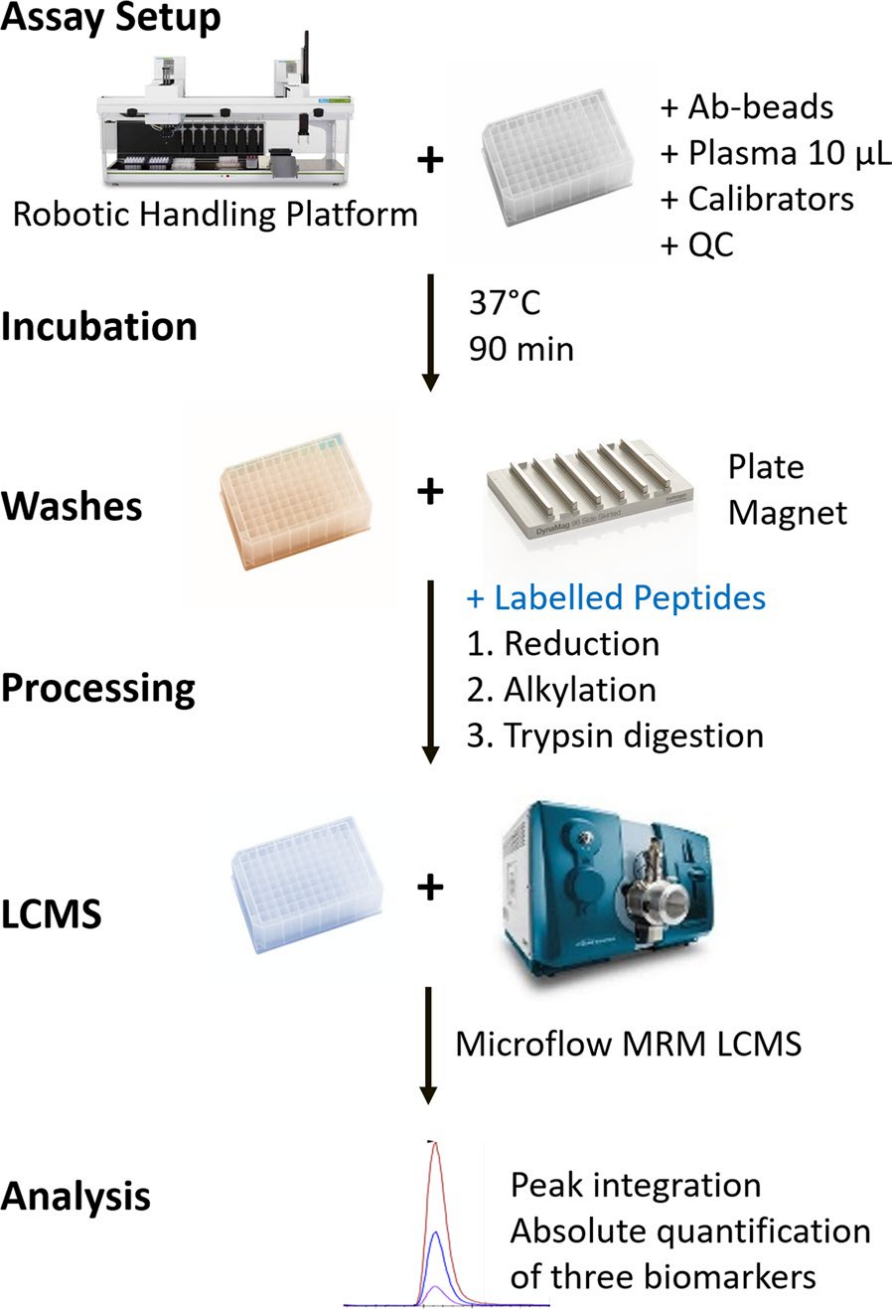
Optimised workflow for immunoaffinity assay for detection of three biomarkers simultaneously in plasma

The conversion of the PromarkerD assay to an immunoaffinity based mass spectrometry test necessitated specific antibodies for each of the three biomarker proteins (CD5L, APOA4, and IBP3). The antibodies were separately coupled to activated beads and tested for their ability to bind firstly purified recombinant protein and then the biomarker proteins in plasma. This process was optimised to provide a reproducible signal response. The assay was produced in a 96 well format that allowed for the implementation of automated processing on a robotic handling platform for liquid handling steps. The magnetic beads enabled removal of solutions easily and efficiently with no meaningful loss of bead material. The LCMS portion of the analysis was transformed from the original nano LCMS (400 nL/min) 90 min run length to a rapid 8 min run using a flow rate of 5 µL/min into the ion source with a source needle of 50 micron internal diameter to provide spray stability at this flow rate. This method is shown in Fig. 1.

The linearity, limits of detection (LOD), limits of quantification (LOQ) and range of the assay were determined from six point dilution series of the three biomarkers processed by the assay. For APOA4, the range of quantification was 37.5 to 200 µg/mL with the LOD at 9.40 µg/mL and a linearity R^2^ of 0.983. CD5L had a range of 0.104 to 10.0 µg/mL with a LOD at 0.100 µg/mL and a R^2^ of 0.997. The third biomarker, IBP3 had a range of 0.0104 to 1.00 µg/mL with a LOD of 0.0100 µg/mL and an R^2^ of 0.997. These ranges of quantification covered the range of detected concentrations for plasma samples in this study. The population average level of APOA4 as measured by Verges et al. [22] was 180 µg/mL for healthy individuals and 120 µg/mL for diabetics. The population average level for CD5L as measured by Yamazaki et al. [23] was 5.5 µg/mL (no data for diabetics specifically). The population average for IBP3 as measured by Wennberg et al. [24] was 3.5 µg/mL for the population as a whole and 3.1 µg/mL for diabetics specifically.[These population values across all 3 biomarkers were measured by ELISA so may not be directly comparable to the current immunoaffinity LCMS values and ranges obtained.]

A parallelism experiment to test the response of the recombinant protein against the endogenous protein in a plasma sample was performed using the recombinant protein in PBS against a reference plasma diluted with human serum albumin across the range of the assay. The slope of each of the plots was compared between the two dilutions with an APOA4 PBS slope of 0.024 (R^2^ = 0.99) versus a slope of 0.025 (R^2^ = 0.99) for APOA4 in plasma. The slope of CD5L in PBS was 0.102 (R^2^ = 0.99) with the slope of CD5L in plasma at 0.099 (R^2^ = 0.99). For IBP3 in PBS the slope was 0.9245 (R^2^ = 0.98) and in plasma a slope of 0.830 (R^2^ = 0.98). For all 3 biomarkers there was parallelism observed between the solutions of recombinant versus endogenous protein with no significant difference across the range of observed dilutions.

Testing of the robustness of the PromarkerD immunoaffinity MS assay was performed by temperature stability testing and intra/interday comparison of a reference plasma. The temperature stability of the biomarker proteins was assessed using replicates of 3 independent plasma samples that were left for 1 h or 24 h at 4 °C or RT before processing and analysis. Multiple freeze thaw cycles were also tested on replicates of the 3 plasma samples as well as the stability of processed samples for 24 h at 4 °C to simulate delays in injection of processed samples. The results of these stability tests are shown in Table 1 with the full data used to generate Table 1 shown in Additional file 2.

**Table 1:** Immunoaffinity assay temperature, freeze-thaw, and extract, stability

	4 °C		RT		24 h		Freeze–thaw		Freeze–thaw		Extract
(N = 3)	24 h vs 1 h		24 h vs 1 h		RT vs 4 °C		2 vs 1		3 vs 1		24 h, 4 °C, (N = 86),
							Freeze–thaw		Freeze–thaw		Autosampler
Protein	Accuracy	Precision (% CV)	Accuracy	Precision (% CV)	Accuracy	Precision (% CV)	Accuracy	Precision (% CV)	Accuracy	Precision (% CV)	Precision (% CV)
APOA4	101.6	10.0	92.6	7.4	84.0	3.6	107.4	13.3	100.8	19.0	0.9
CD5L	118.4	8.6	103.8	10.1	95.4	14.5	106.7	5.7	106.1	14.8	8.5
IBP3	95.3	13.9	91.8	5.5	99.6	4.3	101.1	11.9	95.2	15.1	2.1

Accuracy data are expressed as average percentage (N = 3) compared to control value equalling 100%. Precision data are the average % CV of the replicate comparisons

The stability of the biomarkers to sample handling met the acceptance criteria of < 20% CV based on “FDA Bioanalytical Method Validation Guidance” for ligand binding assays [25], with the accuracy and precision of the biomarker measurements within specifications.

Aliquots of the same reference plasma sample were tested with the assay for intraday (N = 4) and interday (N = 20) comparisons shown in Table 2.

**Table 2:** Immunoaffinity assay intraday and interday variability

Protein	Intra-day (N = 4)		Inter-day (N = 20)	
	Concentration	Precision	Concentration	Precision
	(µg/mL)	(% CV)	(µg/mL)	(% CV)
APOA4	79.4 ± 7.5	9.4	75.8 ± 7.1	9.4
CD5L	2.77 ± 0.21	7.6	2.50 ± 0.24	9.8
IGFBP3	0.27 ± 0.01	5.6	0.29 ± 0.03	10.5

Concentration data are mean ± SD. Precision based on the average % CV

The measurements of either intraday or interday variability across 20 separate assays spanning a timeframe of 2 months were all < 11% CV, well within FDA bioanalytical guidelines [25] for ligand binding assays of < 20% CV. These results demonstrate the robustness of the assay and reproducibility of measurement.

**Fig. 2:**
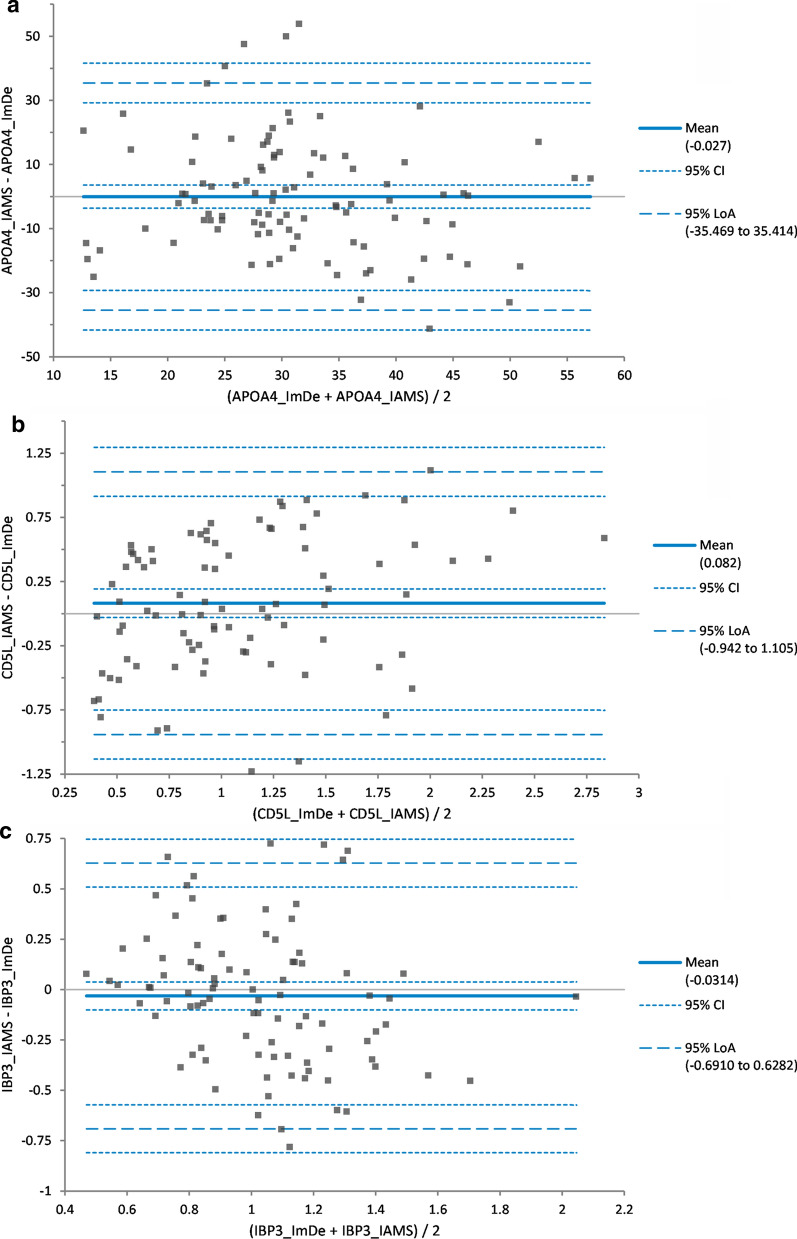
Difference plots for original immunodepletion (ImDe) method and new immunoaffinity (IAMS) method biomarker concentrations. Panel A is for APOA4, panel B for CD5L and panel C for IBP3. These difference plots show the agreement between methods for each biomarker, after IAMS concentrations were adjusted based on the mean bias determined from Bland Altman plot analysis. LoA is Limits of Agreement. 95%CI is the 95% Confidence Interval

**Fig. 3:**
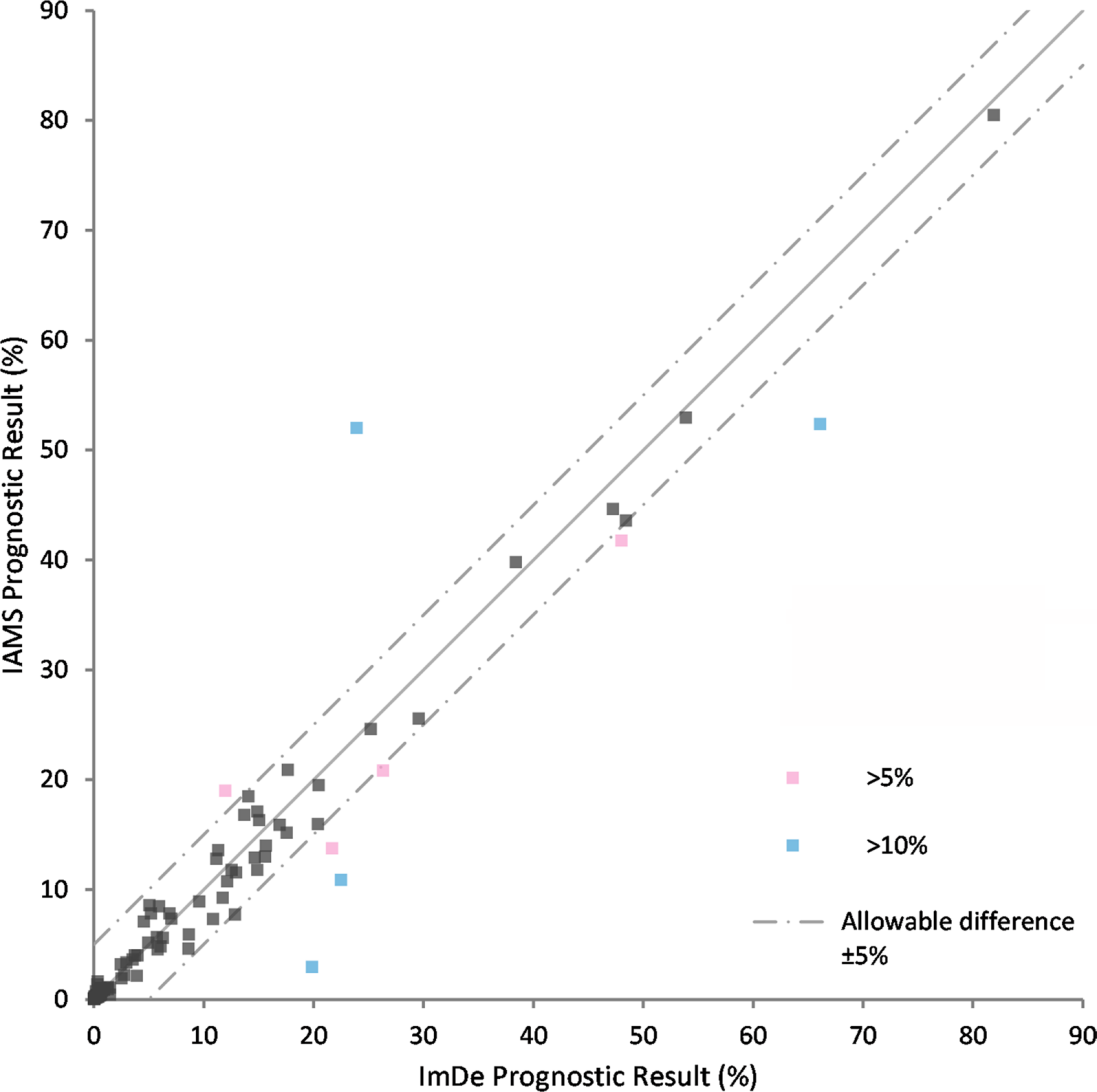
Method comparison. Scatter plot of the correlation between the original immunodepletion (ImDe) method and the new immunoaffinity (IAMS) method PromarkerD risk scores. Generated after individual biomarker Bland–Altman analyses between the methods. Slope of the line of equality is 1

The published PromarkerD risk prediction algorithm was derived using an immunodepletion workflow, hence it was necessary to test the two forms of the assay against each other to ensure they produced the same results across a range of samples. Consequently, aliquots of 100 plasma samples were analysed by both assay methods to determine the target biomarker concentrations. After a Bland Altman analysis of each protein (Additional file 3), immunoaffinity biomarker concentrations were adjusted (Fig. 2, Additional file 4) and the PromarkerD risk score was calculated and the results compared between the two methods, with a coefficient of correlation, R = 0.96 achieved (Fig. 3, Additional file 4. From the comparison, 92% of the subjects had acceptable differences (< 5%) between the methods.

To first assess the capability of running this mass spectrometry based diagnostic test in different laboratories a simple experiment was performed. A batch of 8 unique plasma samples were processed with the original immunodepletion method [14] at the development laboratory and the processed sample was then split and run at a second site with equivalent instrumentation (the same LCMS method on a Sciex 4000 QTRAP versus a Sciex 5500 QTRAP). Across the samples, the results showed for APOA4 the mean difference in concentration between the two sites was 4% with a CV of the differences of 3.3%. For CD5L the mean difference was 1% with a CV of 6.0% and for IBP3 a mean difference of 6% with a CV of 8.9% (Data not shown). This demonstrated the ability to run the same set of transitions for the same biomarkers from the same samples on different instruments and provide precise results.

**Fig. 4:**
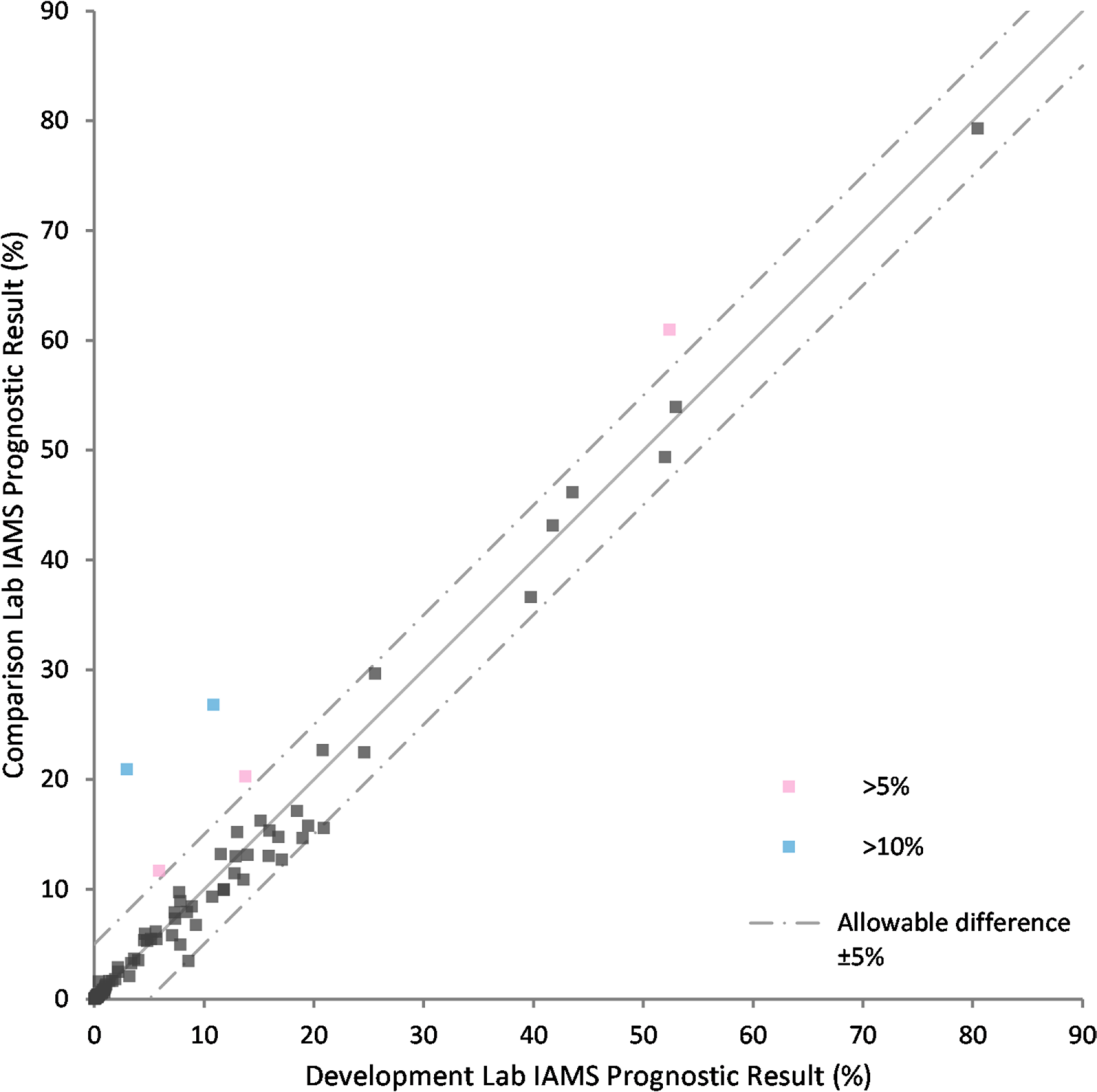
Inter-laboratory comparison. Scatter plot of the correlation between the development laboratory and an independent comparison laboratory with the immunoaffinity method (IAMS) after individual biomarker Bland–Altman analyses between the laboratories. Slope of the line of equality is 1

A full inter-laboratory crossover experiment was then performed following transfer of the technology to a third site. Samples from 100 patients were tested in each laboratory to determine the biomarker concentrations. The same batch of antibody-beads and labelled peptides were used across both laboratories. Each laboratory followed the same immunoaffinity sample preparation method with all standards, calibrators and reagents (except antibody-beads and labelled peptides) made independently to the same formula. The analysis used comparable but different LCMS systems. A Bland Altman analysis of the biomarker concentrations was then performed between the results from each laboratory (Additional files 5 and 6 (panels A,B,C). The resulting PromarkerD risk score comparison between the laboratories showed a high degree of correlation, R = 0.98 (Fig. 4). From the comparison, 95% of the subjects had acceptable differences (< 5%) between the methods.

## Discussion

The translation of a discovered protein biomarker to a clinical grade assay capable of measuring thousands of samples in a reproducible, robust and efficient manner is not a trivial exercise. There are many steps that involve optimisation from the sample handling and processing as well as the particular analysis in a liquid chromatography mass spectrometry system. This study has described the process of converting what was, from processing to data analysis, a week long procedure into a two-day assay output for a batch of samples. The processing method was transformed from a labour intensive and expensive immunodepletion workflow that targeted three biomarkers from within a still complex sub-proteome of plasma, to an elegant multiplexing immunoaffinity capture based methodology providing a clean and selective sample comprising only the biomarkers of interest.

This immunoaffinity method could be automated in a 96 well format with minimal human intervention and the attendant advantages such robotic handling provides. The nature of the immunoaffinity capture of just the target biomarkers provides a significantly cleaner sample than an immunodepletion strategy where the sample for analysis is the entire plasma proteome minus only the most abundant plasma proteins. This fundamental difference in the methods is the most likely source of bias found between the measured biomarker concentrations following Bland Altman analysis. This extra sample cleanliness allowed the application of a more stable low microlitre per minute flow rate which retained sufficient sensitivity of detection. The use of a more robust ion source (compared to the nanoflow source) with constant spray stability also offers increased uptime of the system as a whole when analysing large numbers of samples. The 8 min chromatography runtime is more compatible with clinical mass spectrometry methods of small molecule drugs, for instance, and is essential for an assay that would have high sample capacity requirements with quick turnaround times.

An essential test of the viability of a clinical assay is the stability of the analytes under expected or potential conditions that might influence the processing or detection characteristics. The temperature and time variables tested for this assay demonstrated the stable behaviour of the biomarkers under these conditions. Any assay for a clinical application to be employed over an extended period of time needs to be reproducible, both intraday and interday, as this assay demonstrated.

The newly developed clinical assay [26] was compared to its precursor research grade parent, and also tested against itself in an independent laboratory. Both sets of data provided high correlation of the PromarkerD output risk score for progression of diabetic kidney disease. The technology transfer to a second independent laboratory and the independent analysis of the same set of samples are also steps required of an assay seeking to be clinically viable in multiple testing sites. This transfer illustrated the robustness of the new protocol to run such an assay.

## Conclusions

While mass spectrometry based protein biomarker assays have not become mainstream alternatives to ELISA based approaches as yet, the discovery of multi-protein biomarker panels means a multiplexing capability, as easily attainable with targeted mass spectrometry, is a viable strategy. This iteration of a diabetic kidney disease prognostic assay is platform-independent, showing a high-degree of correlation between two independent laboratories as well as between the immunoaffinity and precursor immunodepletion versions. The PromarkerD assay has already demonstrated clinical validity and here demonstrates the capability of immunoaffinity selectivity coupled to targeted mass spectrometry detection, to be a powerful combination in the clinical assay space. This combination means PromarkerD has the potential to support clinical decision-making by identifying at-risk patients for earlier intervention and monitoring of diabetic kidney disease progression, with the potential for improved patient outcomes.

## Supplementary information

**Additional file 1. **Transition settings for mass spectrometry analysis.

**Additional file 2. **Biomarker concentrations for APOA4, CD5L and IBP3 for stability experiments. [Summary of data is Table 1 in the manuscript].

**Additional file 3. **Bland Altman plots of biomarker concentrations for original immunodepletion (Imde) and new immunoaffinity (IAMS) method. A: APOA4 comparison, B: CD5L comparison, C: IBP3 comparison, D:CD5L comparison with regression based 95% limits of agreement, E: IBP3 comparison with regression based 95% limits of agreement.

**Additional file 4. **Biomarker concentrations for APOA4, CD5L and IBP3 for original immunodepletion (Imde) and new immunoaffinity (IAMS) method before and after Bland Altman adjustment. The PromarkerD risk scores are shown for both the ImDe and IAMS methods. Data is used for Figures 2 and 3.

**Additional file 5. **Biomarker concentrations for APOA4, CD5L and IBP3 for development laboratory and comparison laboratory before and after Bland Altman adjustment. The PromarkerD risk scores are shown for both laboratories. Data is used in Figure 4.

**Additional file 6. **Bland Altman plots of biomarker concentrations for development and comparison laboratory before adjustment. A: APOA4 comparison, B: CD5L comparison, C: IBP3 comparison.

## Data Availability

All data generated or analysed during this study are included in this published article and its additional files.

## Abbreviations

Ab-beads: Magnetic beads conjugated with monoclonal antibodies to the biomarker proteins
ACR: Albumin:creatinine ratio
APOA4: Apolipoprotein A4
CD5L: CD5 antigen-like
CKD: Chromic kidney disease
CV: Coefficient of variation
DKD: Diabetic kidney disease
eGFR: Estimated glomerular filtration rate
EDTA: Ethylenediaminetetraacetic acid
ELISA: Enzyme-linked immunosorbent assay
FDA: Food and Drug Administration
FDS: Fremantle Diabetes Study
IAM: Iodoacetamide
IAMS: Immunoaffinity mass spectrometry
IBP3: Insulin-like growth factor-binding protein 3
ImDe: Immunodepletion
LCMS: Liquid chromatography mass spectrometry
LoA: Limits of agreement
LOD: Limits of detection
LOQ: Limit of quantification
MRM: Multiple reaction monitoring
N: Number
NaCl: Sodium chloride
PBS: Phosphate buffered saline
QC: Quality control
R: Coefficient of correlation
R^2^: Coefficient of determination
RT: Room temperature
S/N: Signal to noise
TCEP: Tris(2-carboxyethyl)phosphine hydrochloride
TEAB: Triethylammonium bicarbonate
