# Supplementary figures and images for: A robust multiplex immunoaffinity mass spectrometry assay (PromarkerD) for clinical prediction of diabetic kidney disease
Source: Clin Proteomics. 2020 Oct 20;17:37. doi: 10.1186/s12014-020-09302-w (PMC7576806; doi:10.1186/s12014-020-09302-w)

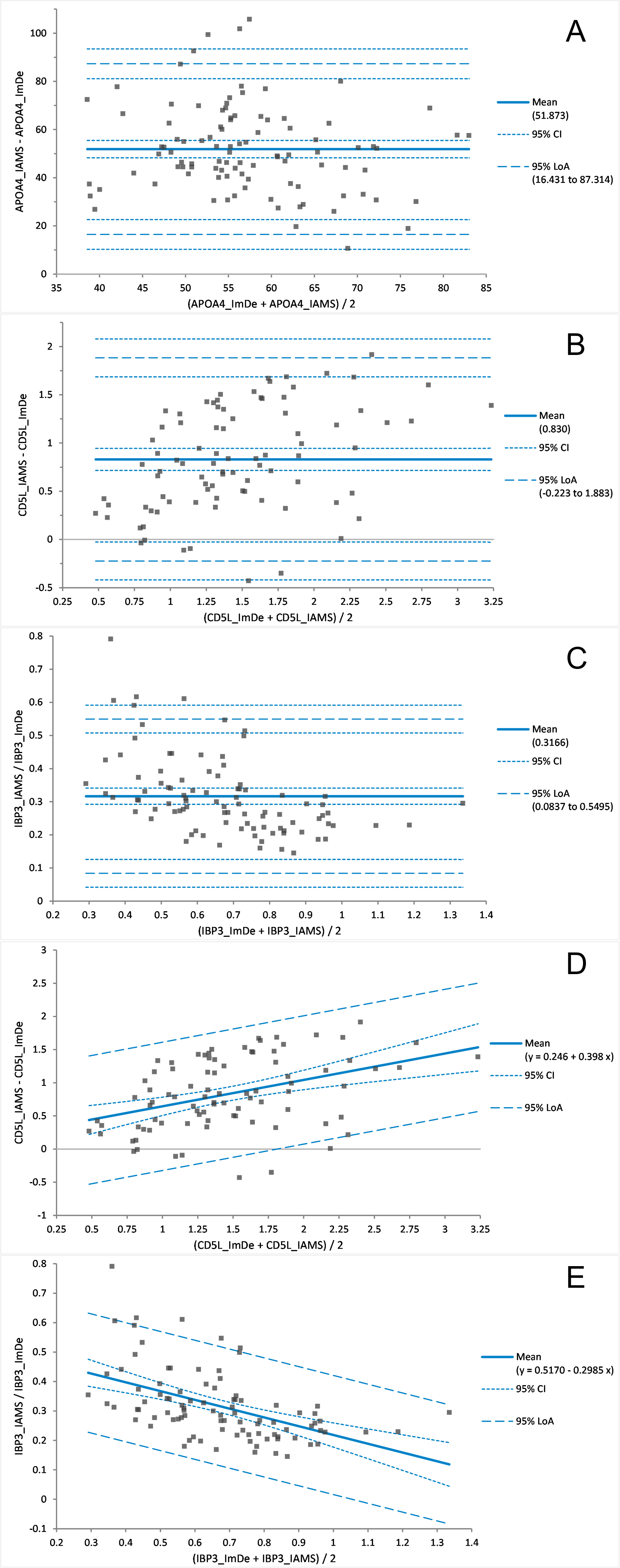

Supplement: Supplementary file 3 — Additional file 3. Bland Altman plots of biomarker concentrations for original immunodepletion (Imde) and new immunoaffinity (IAMS) method. A: APOA4 comparison, B: CD5L comparison, C: IBP3 comparison, D:CD5L comparison with regression based 95% limits of agreement, E: IBP3 comparison with regression based 95% limits of agreement. [file 12014_2020_9302_MOESM3_ESM.png]

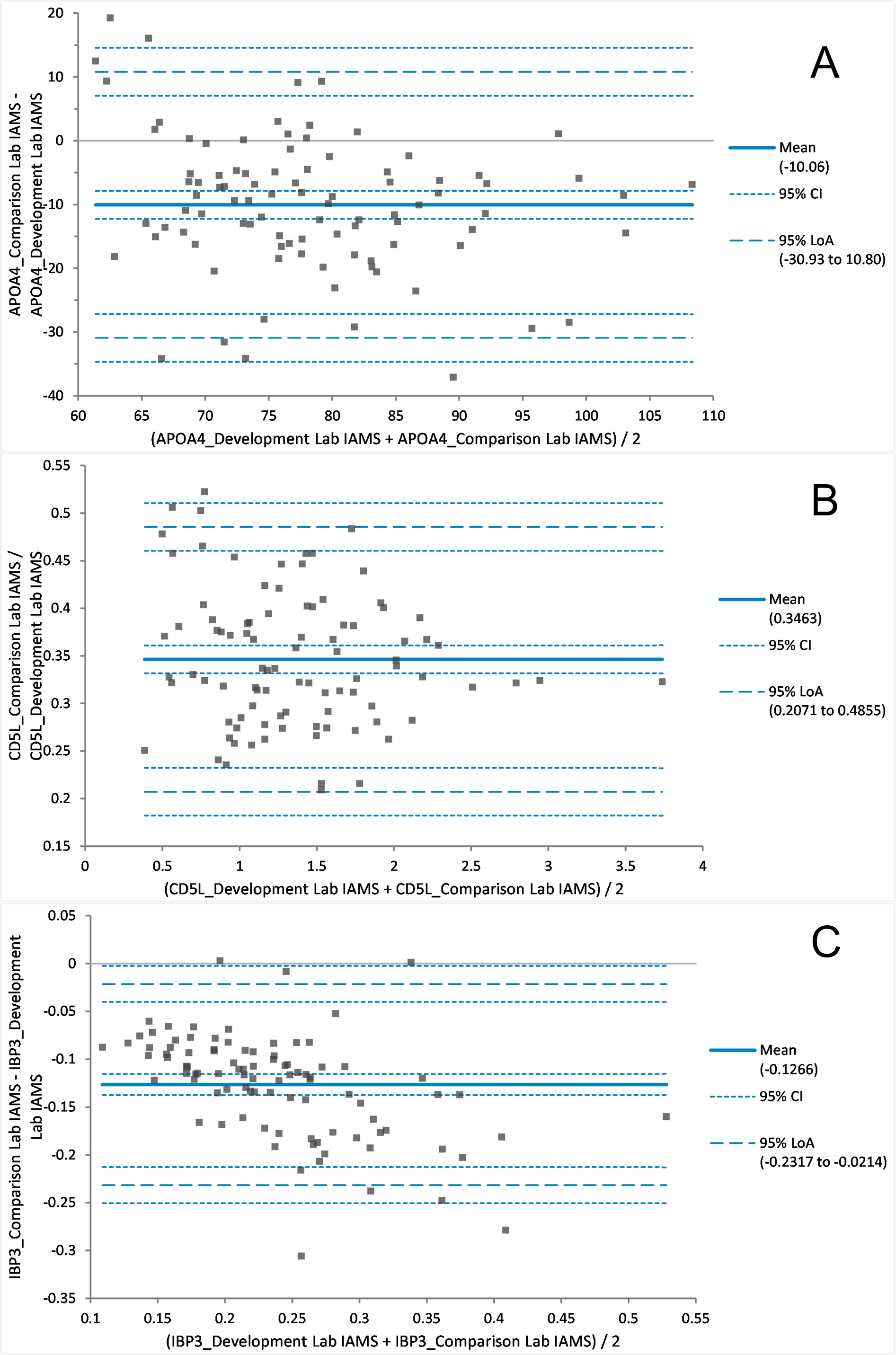

Supplement: Supplementary file 6 — Additional file 6. Bland Altman plots of biomarker concentrations for development and comparison laboratory before adjustment. A: APOA4 comparison, B: CD5L comparison, C: IBP3 comparison. [file 12014_2020_9302_MOESM6_ESM.png]
